# Supplementary material for: Photoreceptor glucose metabolism determines normal retinal vascular growth
Source: EMBO Mol Med. 2017 Nov 27;10(1):76–90. doi: 10.15252/emmm.201707966 (PMC5760850; doi:10.15252/emmm.201707966)
Supplement: Supplementary file 1 — Expanded View Figures PDF [file EMMM-10-76-s001.pdf]

Expanded View Figures

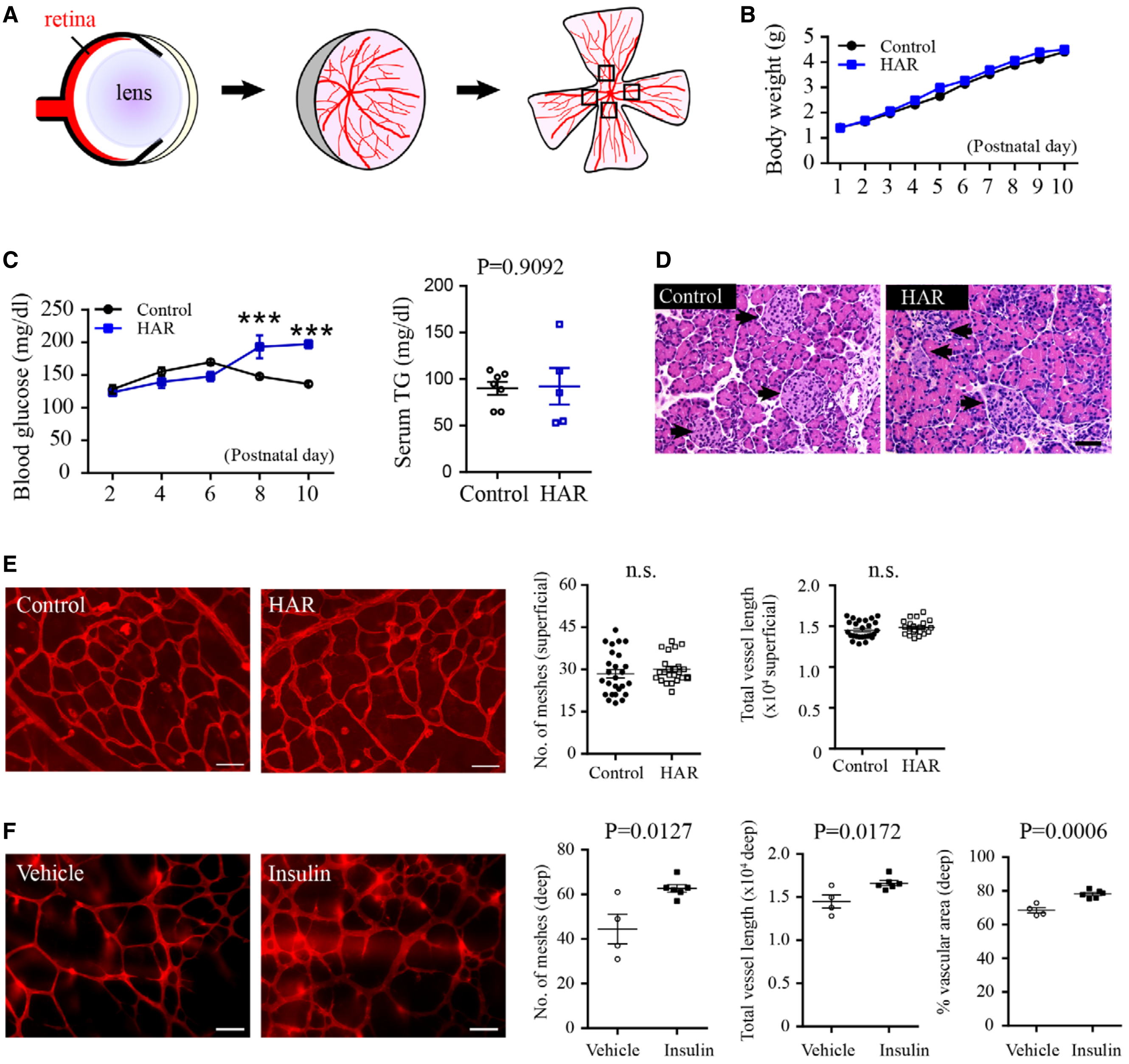

Figure EV1. Schematic of retinal dissection; the superficial retinal vasculature was quantified in hyperglycemia-associated retinopathy.

The mouse neonates received i.p. injection of 50 mg kg^{-1} STZ (diluted in PBS) from postnatal (P) days 1–9 (related to Fig 2).

A Schematics of images taken for quantification of vascular layers.

B Body weight and body growth starting from birth between control and mice induced for hyperglycemia-associated retinopathy (HAR). n = 17–24/group.

C Blood glucose levels starting from P2 to P10 (n = 7–19/group, ANOVA, $^{***}P < 0.001$) and serum triglyceride levels at P8 (n = 5–7/group, unpaired t-test) in control and HAR mice. Data presented as mean ± SEM.

D At P10, the pancreas was stained with H&E. STZ treatment disrupted the pancreatic islet structure. The pancreatic islet structure (arrows) is distorted in STZ-induced hyperglycemia-associated retinopathy group. Scale bar: 50 μm.

E Formation of the superficial vascular layer in control and hyperglycemia-associated retinopathy retinas. Scale bar: 50 μm. n = 23–26/group. Unpaired t-test. n.s., not significant. Data presented as mean ± SEM.

F Deep retinal vascular formation in WT HAR mice with either insulin or vehicle treatment in littermates. Scale bar: 50 μm. n = 4–6/group. Unpaired t-test. Data presented as mean ± SEM.

 © 2017 The Authors

Figure EV2. There was no direct effect of STZ on retinal vasculature; superficial vascular network was quantified with APN deficiency or with activation of APN receptors (related to Fig 3).

A No direct toxicity of STZ on retinal vascular development. The mouse neonates at P1 received a single intravitreous injection of STZ (1 µg/eye), and the contralateral eye was injected with PBS (vehicle). At P10, there were no significant changes in the number of meshes, total vessel length, or % of vascular coverage in the superficial or deep retinal vascular layers. *n* = 5–6 retinas/group. Unpaired *t*-test. n.s., not significant.

B Superficial and deep retinal vascular formation under normoglycemia in WT and *Apn*$^{-/-}$ retinas. *n* = 19–24 retinas/group.

C Superficial vascular network formation in *Apn*$^{-/-}$ versus WT hyperglycemia-associated retinopathy mice. *n* = 21–23 retinas/group.

D Superficial vascular network formation in msAPN- versus vehicle-treated WT hyperglycemic mice. *n* = 7–8 retinas/group.

E Superficial vascular network formation in adipoRon- versus vehicle-treated WT hyperglycemic mice at P10. *n* = 8–14 retinas/group.

Data information: Unpaired *t*-test (B, C, E), Mann–Whitney test (D); n.s., not significant. Data presented as mean ± SEM (A–E).

© 2017 The Authors

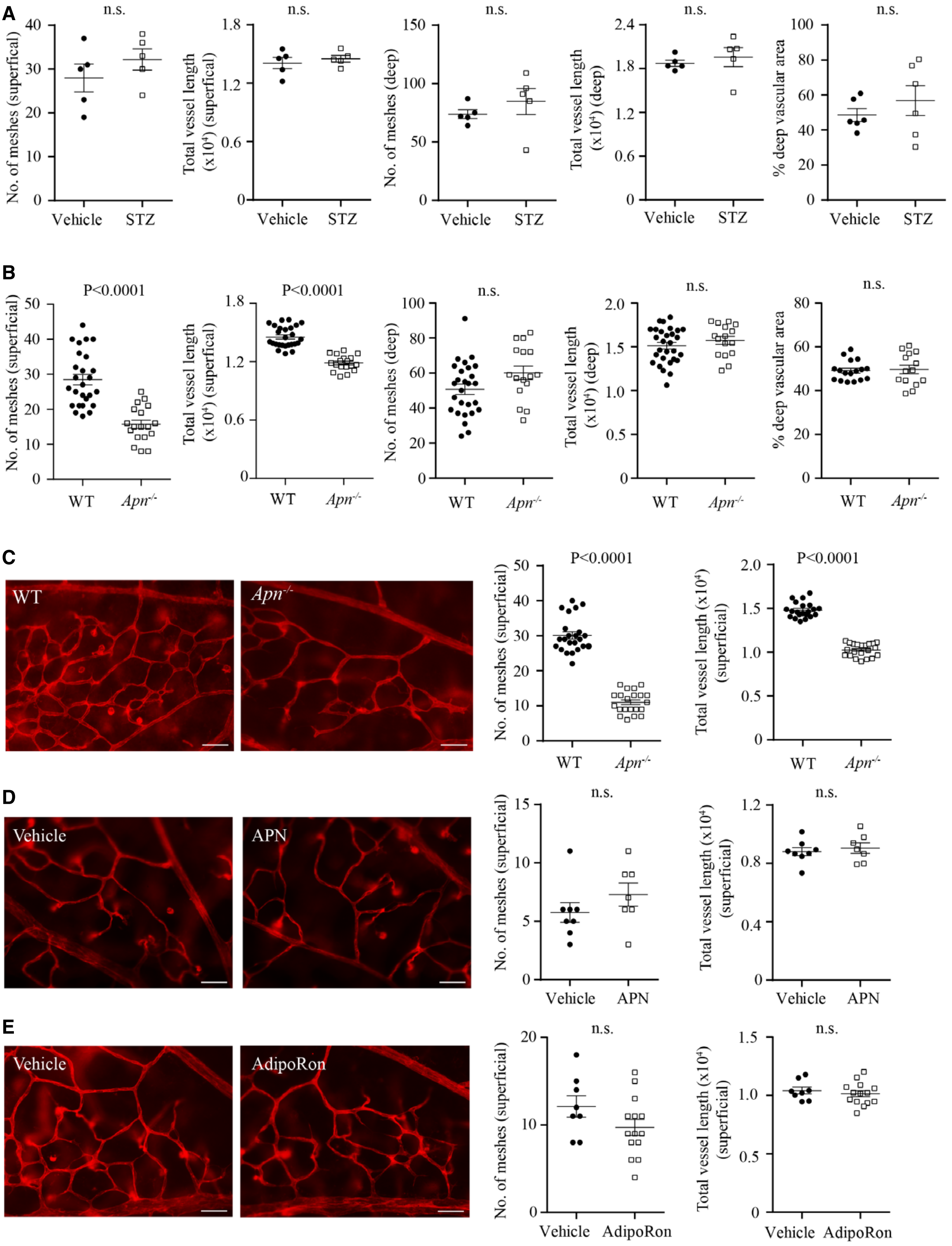

Figure EV2.

 © 2017 The Authors

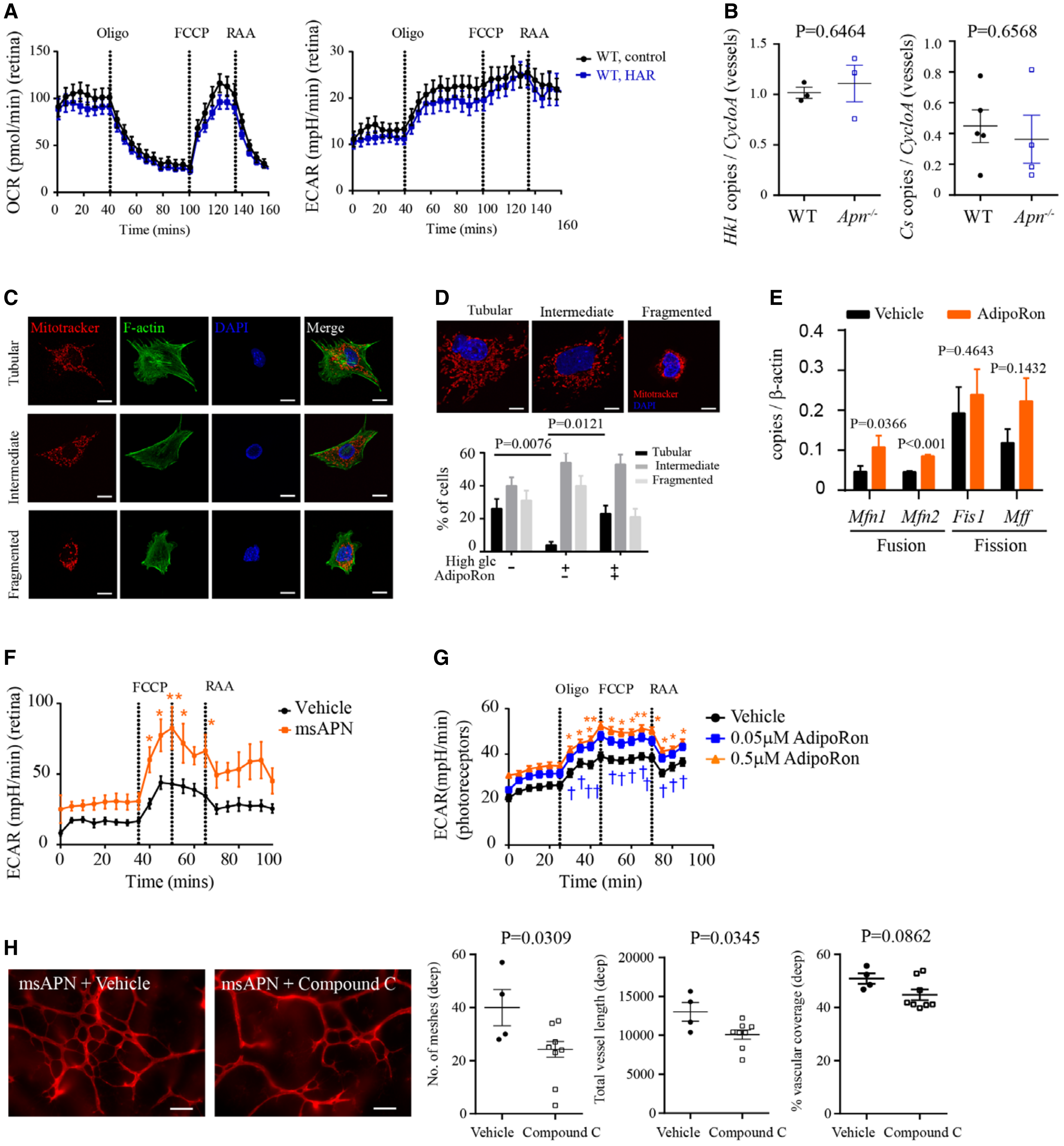
A
Oligo
FCCP
RAA
OCR (pmol/min) (retina)
Time (mins)
ECAR (mpH/min) (retina)
WT, control
WT, HAR
B
P=0.6464
Hk1 copies / CycloA (vessels)
WT
Apn-/-
P=0.6568
Cs copies / CycloA (vessels)
C
Mitotracker
F-actin
DAPI
Merge
Tubular
Intermediate
Fragmented
D
Tubular
Intermediate
Fragmented
P=0.0076
P=0.0121
% of cells
High glc
AdipoRon
E
Vehicle
AdipoRon
copies / β-actin
P=0.0366
P<0.001
P=0.4643
P=0.1432
Mfn1
Mfn2
Fis1
Mff
Fusion
Fission
F
FCCP
RAA
Vehicle
msAPN
ECAR (mpH/min) (retina)
Time (mins)
G
Oligo
FCCP
RAA
Vehicle
0.05μM AdipoRon
0.5μM AdipoRon
ECAR(mpH/min) (photoreceptors)
Time (min)
H
msAPN + Vehicle
msAPN + Compound C
P=0.0309
No. of meshes (deep)
P=0.0345
Total vessel length (deep)
P=0.0862
% vascular coverage (deep)
Vehicle
Compound C

Figure EV3.

◀

Figure EV3. Activation of the APN pathway improved photoreceptor mitochondrial activity (related to Fig 4).

- A Oxygen consumption rate (OCR) and extracellular acidification rate (ECAR) of WT control and HAR P10 retinas. n = 5–6 animals (24–32 punches) per group.
- B APN deficiency did not affect retinal metabolic enzyme mRNA in deep retinal blood vessels. qRT–PCR of *Hk1* and *Cs* in LCM deep blood vessels in *Apn*$^{-/-}$ versus WT HAR mice. n = 3 pooled retinas/group. Unpaired t-test.
- C Mitochondrial and cytoskeleton morphology in 661W cells. Cell was stained with MitoTracker for mitochondria (red), F-actin for cytoskeleton (green), and DAPI for nucleus (blue). Images were taken under 630× magnification. Scale bar, 10 μm.
- D Mitochondrial morphology (MitoTracker, red) under hyperglycemic condition, in the presence of AdipoRon or vehicle. Images were taken under 630× magnification. Scale bar, 10 μm. The number of cells within each category of mitochondrial morphology (tubular, intermediate, and fragmented) was counted and represented as a percentage of the total number of cells. n = 30 images/group. Kruskal–Wallis test.
- E qRT–PCR of mitochondrial fusion (*Mfn1, Mfn2*) and fission (*Fis1, Mff*) proteins in AdipoRon- versus vehicle-treated 661W cells. n = 7–9/group. Unpaired t-test (*Mfn1, Mfn2, Mff*); Mann–Whitney test (*Fis1*).
- F Extracellular acidification rate (ECAR) of WT retinas treated with msAPN or vehicle (PBS). n = 6 animals (12 punches) per group. ANOVA, $^*P < 0.05$, $^{**}P < 0.01$.
- G ECAR of 661W cells treated with AdipoRon or vehicle. n = 10 per group. ANOVA, $^*P < 0.05$, $^{**}P < 0.01$ (0.5 μM AdipoRon versus vehicle); $^{\dagger}P < 0.05$, $^{\dagger\dagger}P < 0.01$ (0.05 μM AdipoRon versus vehicle).
- H Deep retinal vascular formation in WT HAR mice co-treated with msAPN (0.6 μg g^{-1}) and Compound C (AMPK inhibitor, 2 μg g^{-1}) or vehicle from P7 to P9. n = 4–8 per group. Scale bar: 50 μm. Unpaired t-test.

Data information: Data presented as mean ± SEM (A, B, D–H).

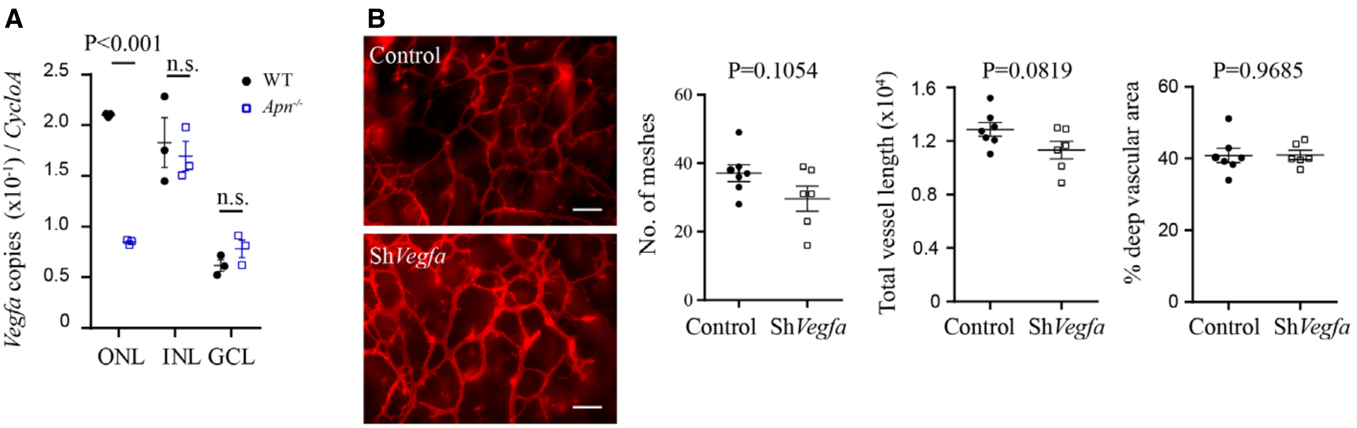

Figure EV4. Photoreceptor-derived VEGFA was modulated by the APN pathway but not contributed to normal deep blood vessel growth (related to Fig 4).

- A *Vegfa* mRNA expression in retinal neuronal layers (ONL, INL, GCL) of *Apn*$^{-/-}$ versus WT P10 HAR retinas (LCM and qRT–PCR). n = 3 pooled retinas/group. ANOVA. n.s., not significant. Data presented as mean ± SEM.
- B Representative images of deep retinal vascular network and quantification in AAV2-hRK-*Vegfa*-GFP versus AAV2-hRK-GFP (control) virus subretinally injected in WT mice. Retinas were examined at P10. Scale bar, 50 μm. n = 6–7 per group. Unpaired t-test. Data presented as mean ± SEM.

 © 2017 The Authors
